# Synergistic Impacts of *Alpinia oxyphylla* Seed Extract and Allopurinol against Experimental Hyperuricemia

**DOI:** 10.1155/2022/2824535

**Published:** 2022-06-11

**Authors:** Yoon-Young Sung, Dong-Seon Kim

**Affiliations:** KM Science Research Division, Korea Institute of Oriental Medicine, 1672 Yuseong-daero, Yuseong-gu, Daejeon 34054, Republic of Korea

## Abstract

In traditional medicine, *Alpinia oxyphylla* Miquel seed has been used to treat gout and hyperuricemia-related symptoms by enhancing kidney functions. Allopurinol is the most commonly used drug to treat hyperuricemia; however, the drug has many adverse effects. Combining allopurinol with another compound could reduce the need for high doses and result in improved safety. We investigated the possible synergistic effects of *Alpinia oxyphylla* seed extract (AE) and allopurinol in decreasing urate concentrations in rats with potassium oxonate-induced hyperuricemia. This study evaluated the effects of allopurinol combined with AE on levels of serum urate, blood urea nitrogen (BUN), and creatinine in a hyperuricemic rat model. The effects of allopurinol plus AE on xanthine oxidase (XOD) activity and urate uptake were measured. The concomitant administration of allopurinol and AE normalized serum urate and reduced BUN and creatinine. The attenuation of hyperuricemia-induced impaired kidney function was related to downregulation of renal urate transporter 1 and upregulation of renal organic anion transporter 1, with inhibition of serum and hepatic XOD activities. The antihyperuricemic effects of allopurinol were enhanced when combined with AE. These results suggested that the combined use of allopurinol and AE may have clinical efficacy in treating hyperuricemia.

## 1. Introduction

Excess urate in the blood is an important risk factor for the development of gout, diabetes, and kidney disease [1–3]. Hyperuricemia is an abnormal condition characterized by increased levels of serum urate. Gout is a very common, painful inflammatory arthritis and is characterized by metabolic disorders involving hyperuricemia and precipitation of monosodium urate crystals in tissues, such as the kidneys and joints because of an imbalance in synthesis, uptake, or excretion of urate [4].

The biosynthesis of urate in the liver is mediated via xanthine oxidase (XOD) in two stages (conversion of hypoxanthine to xanthine and oxidation of xanthine to urate) of urate generation [5]. Reabsorption and secretion of urate are main factors contributing to circulating high levels of urate and are mainly mediated by various anion transporters containing urate transporter 1 (URAT1), glucose transporter 9 (GLUT9), and organic anion transporters (OAT) 1 and 3 in the renal proximal tubules [6]. XOD and these transporters are thus essential targets in treating hyperuricemia.

Allopurinol is a common urate-lowering medication used to treat gout and hyperuricemia [7]. As a purine inhibitor of XOD, it inhibits urate synthesis as the final product of purine catabolism [8]. However, clinical trials have shown that >50% of patients could not maintain a reduction in serum urate levels at the most commonly used dose (300 mg) [9]. In some patients, allopurinol is less effective and induces serious side effects, including pruritus, skin rash, fever, hepatitis, renal toxicity, and leukocytosis with eosinophilia [10]. Thus, it is clinically desirable to reduce the required high doses and potentiate the effects of allopurinol [1].

The *Alpinia oxyphylla* Miquel (Zingiberaceae family) plant is a well-known traditional herbal medicine in Asia. The dried seed is used to treat diuresis, enuresis, intestinal disorders, and chronic glomerulonephritis [11]. In traditional medicine, this seed has been used to treat symptoms of gout and hyperuricemia by enhancing kidney functions [12–15]. The kidney is a key organ for uric acid reabsorption and secretion. In pharmacological studies, the seed has been shown to have a variety of effects, including neuroprotective, antitumorigenic, and antiallergic activities [16, 17]. We previously demonstrated that *A. oxyphylla* extract and its constituent nootkatone reduced serum urate concentrations in a potassium oxonate- (PO-) induced hyperuricemia rat by enhancing urate excretion [15]. In this study, we showed that *A. oxyphylla* regulated URAT1 and OAT1, the urate transporters responsible for the reabsorption and secretion of urate in the kidneys. Combining *A. oxyphylla* with the XOD inhibitor, allopurinol, may provide a dual mechanism for lowering serum uric acid concentrations via increased urate excretion and reduced urate production. Furthermore, we determined whether *A. oxyphylla* potentiated the antihyperuricemic effects of allopurinol in a hyperuricemic rat model induced by PO.

## 2. Materials and Methods

### 2.1. Preparation of a Plant Extract for Combination with Allopurinol

*A. oxyphylla* seed extract was provided by Kwangdong Pharm Co. Ltd. (Seoul, Republic of Korea), and a voucher specimen (No. P008) was stored at the Korea Institute of Oriental Medicine (KIOM), Daejeon, Republic of Korea. The plant was authenticated based on the macroscopic characteristics provided by the Classification and Identification Committee of KIOM. A preliminary investigation of various extracts (water, 30% ethanol, 50% ethanol, and 70% ethanol) of *A. oxyphylla* seeds showed that a 30% ethanolic extract had the most antihyperuricemic activity [15]. Thus, the dried *A. oxyphylla* (250 kg) seed was extracted with 30% ethanol solution for 9 h at 75°C. After filtration, the residue was reextracted with an aqueous solution for 9 h at 95°C. After filtration, the combined filtrates were concentrated under reduced pressure and sterilized at 95°C for 1 h. Allopurinol was obtained from Sigma-Aldrich (St. Louis, MO, USA). Allopurinol and *A. oxyphylla* extract were administrated at a 1 : 200 ratio (w/w) for the combination treatment. In our previous study, the concentration of the main component of *A. oxyphylla* seed extract, nootkatone (1.03 ± 0.13 mg/g), was determined by UPLC analysis for standardization [15].

### 2.2. Animals

Male Sprague Dawley rats (7 weeks of age, 200–250 g) were obtained from Orient Bio (Seongnam, Republic of Korea). They were adapted in an air-conditioned animal room at a temperature of 22 ± 1°C and humidity of 50 ± 10%. The rats had *ad libitum* access to food and water. The protocol was approved by the Committee on Animal Care of the KIOM, and animal procedures were permitted in accordance with the Guide for the Care and Use of Laboratory Animals (National Institutes of Health publication number 85-23; 1996 edition).

### 2.3. Hyperuricemia Induction and Treatment

PO, an inhibitor of urate oxidase, was injected intraperitoneally into rats to induce hyperuricemia as described previously [18]. PO was dissolved in a 0.5% carboxymethyl cellulose (CMC)/pH 5.0, 0.1 M sodium acetate solution, and 150 mg/kg PO was injected intraperitoneally into rats, which were maintained for 1 h. The animals were divided into five groups (*n* = 5 each): the normal control group (NC), the PO-treated hyperuricemia group, the PO+1 mg/kg allopurinol group (AP), the PO+200 mg/kg AE group, and the PO + combination of 1 mg/kg AP and 200 mg/kg AE group (AP + AE, 1 : 200 ratio). The doses of AP and AE used in these experiments were determined from preliminary dose-range experiments (Supplementary materials (available here)) and previously published reports [15]. Allopurinol, AP, AE, and AP + AE samples were dissolved in 0.5% CMC solution and administered by oral gavage to rats, 1 h after PO injection. The NC and PO groups received the vehicle (0.5% CMC). The mice were gently handled to minimize the animal's discomfort and were euthanized by anesthesia with zoletil.

### 2.4. Serum Biochemical Analyses

Blood from rats was collected 2 h following AP, AE, or AP + AE administration, and the serum was separated by centrifugation (3,000 × *g*, 15 min, 4°C). Urine samples were collected 2 h later using a metabolic cage following sample administration. Urate levels from serum and urine were determined using a uric acid assay kit (Abcam, Cambridge, UK). Serum interleukin (IL)-1*β* levels were determined using an ELISA assay kit (R&D Systems, Minneapolis, MN, USA). Glutamic oxaloacetic transaminase (GOT), glutamic pyruvic transaminase (GPT), creatinine, and blood urea nitrogen (BUN) in the serum were measured using a biochemical analyzer (Hitachi 7080, Hitachi Co., Tokyo, Japan). Fractional renal clearance of urate (FCU, fractional excretion of urate; FEUA) was calculated by the formula FCU, renal clearance of urate/renal clearance of creatinine = (urine urate × serum creatinine)/(serum urate × urine creatinine) × 100.

### 2.5. In Vivo XOD Inhibition Assay

Serum and liver XOD activities were measured using a commercial xanthine oxidase activity assay kit (Sigma-Aldrich) according to the manufacturer's protocols. Protein concentrations were measured using a DC protein assay (Bio-Rad, Hercules, CA, USA) using bovine serum albumin as the standard to normalize XOD activity. Liver XOD activity was presented as nanomoles of urate formed/min/mg of protein.

### 2.6. Western Blotting

Protein extracts from kidney tissues were homogenized with PRO-PREP protein extraction solution (Intron, Seoul, Republic of Korea) and centrifuged (13,000 rpm, 5 min, 4°C). Protein concentrations were measured using a DC protein assay (Bio-Rad). The proteins (10 *μ*g) were separated using sodium dodecyl sulfate-polyacrylamide gel electrophoresis. After transfer, the membranes were blocked with EzBlock blocking buffer (ATTO, Tokyo, Japan) for 1 h and incubated with URAT1 (MyBioSource, San Diego, CA, USA), *β*-actin, GLUT9, OAT1, OAT3 (Santa Cruz Biotechnology, Dallas, TX, USA), and IL-1*β* (Abcam) antibodies at 4°C overnight. The protein bands were visualized using an ECL detection reagent (Amersham, Marlborough, MA, USA) using ImageQuant LAS 4000 (GE Healthcare Life Sciences, Seoul, Republic of Korea). The band densities from the obtained images were determined by densitometry using Image J1.49 software (National Institutes of Health, Bethesda, MD, USA). Target protein levels were normalized to *β*-actin.

### 2.7. Kidney Histopathological Examination

Kidney tissues were removed and immediately fixed for 1 day in formalin and embedded in paraffin. Each specimen was cut into 6 *μ*m thick sections and stained with hematoxylin and eosin stain. The sections were then visualized using a light microscope at ×100.

### 2.8. Statistical Analysis

Results are expressed as mean ± standard error of the mean (SEM). Significant differences of the results were tested by one-way analysis of variance followed by Tukey's multiple comparison test using Prism 7.0 software (GraphPad, San Diego, CA, USA). Statistical significance was considered as *p* < 0.05.

## 3. Results

### 3.1. Effects of the AP and AE Combination on Uric Acid Concentrations

Previously, to identify the most effective combination among the combinations of allopurinol and AE, experimental preparations were made by combining allopurinol and AE at different proportions of 1 : 100, 2 : 100, and 1 : 200. The combination of allopurinol and AE at a 1 : 200 ratio was the most effective in decreasing serum uric acid levels among the three mixtures tested. Thus, we selected the allopurinol and AE combination at a ratio of 1 : 200 for further studies.

**Figure 1:**
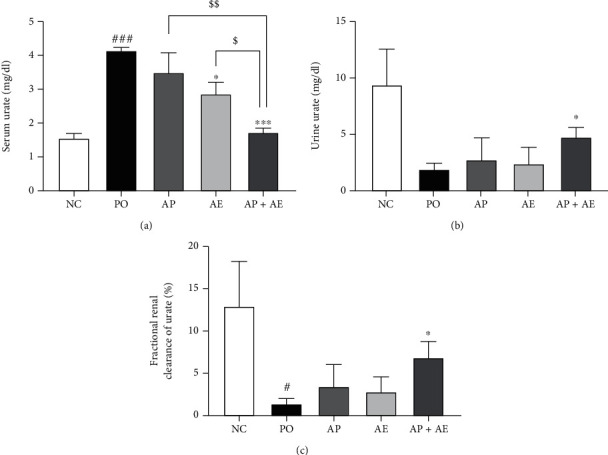
Effects of allopurinol, *Alpinia oxyphylla* seed extract (AE), and their combination on (a) serum urate, (b) urine urate, and (c) fractional renal clearance of urate in hyperuricemic rats. *n* = 5 per group. NC: normal control; PO group: induced with potassium oxonate; AP: allopurinol (1 mg/kg); AE (200 mg/kg); AP + AE: AP (1 mg/kg) plus allopurinol (200 mg/kg). #*p* < 0.05 and ###*p* < 0.001 compared with the NC group; ^∗^*p* < 0.05 and ^∗∗∗^*p* < 0.001 compared with the PO group; $$$*p* < 0.001 compared with the AP or AE groups.

The PO group had significantly higher serum concentrations of urate, when compared with the NC group (Figure 1, *p* < 0.001), indicating effective establishment of the hyperuricemia rat model. Compared with the PO group, serum urate concentrations were not reduced or reduced slightly after treatment with allopurinol (1 mg/kg) or AE (200 mg/kg) alone. However, when a combination of allopurinol and AE (1 : 200) was given, serum urate concentrations were significantly decreased (*p* < 0.05 compared to allopurinol or AE alone). Next, to evaluate the effects of the combination on urate excretion, urine urate levels were determined. Urine urate levels and renal urate excretion as fractional renal clearance of urate were significantly increased by the combination of allopurinol and AE (*p* < 0.05), indicating that the combination of allopurinol and AE increased kidney urate excretion, resulting in decreased serum urate levels in PO-induced hyperuricemia rats.

### 3.2. Effects of AP, AE, and their Combination on Serum GOT, GPT, BUN, and Creatinine Levels

**Figure 2:**
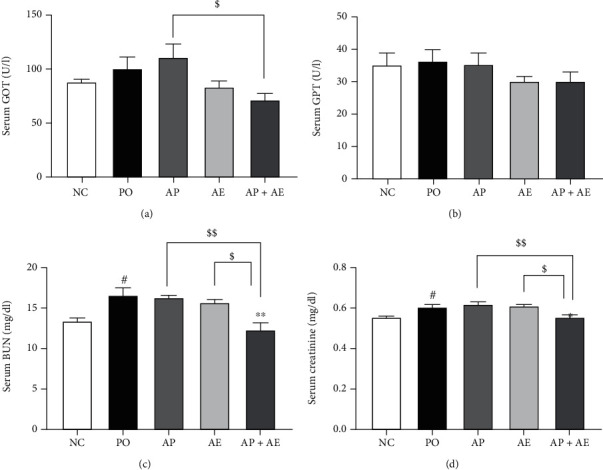
Effects of allopurinol, AE, and their combination on serum glutamic oxaloacetic transaminase (a), glutamic pyruvic transaminase (b), blood urea nitrogen (c), and creatinine (d) in hyperuricemic rats. *n* = 5 per group. NC: normal control; PO group: induced with potassium oxonate; AP: allopurinol (1 mg/kg); AE (200 mg/kg); AP + AE: AP (1 mg/kg) plus allopurinol (200 mg/kg). #*p* < 0.05 compared with the NC group; ^∗^*p* < 0.05 and ^∗∗^*p* < 0.01 compared with the PO group; $*p* < 0.05 and $$*p* < 0.01 compared with the AP or AE groups.

We evaluated serum levels of GOT, GPT, BUN, and creatinine in PO rats. GOT and GPT concentrations did not differ significantly between the other groups; although, GOT levels were decreased by treatment with the combination, when compared with AP alone (Figures 2(a) and 2(b)). The PO group had significantly higher BUN and creatinine concentrations compared to the NC group (*p* < 0.05). AP or AE alone did not affect either serum BUN or creatinine, but the AP and AE combination significantly reduced these levels (*p* < 0.01 and *p* < 0.05, respectively, Figures 2(c) and 2(d)). AE administration significantly enhanced the effects of AP on serum BUN and creatinine levels (*p* < 0.01 and *p* < 0.05, compared to AP or AE alone, respectively). These data indicated that the AP and AE combination decreased PO-induced liver and renal dysfunction.

### 3.3. Effects of the AP and AE Combination on Serum and Hepatic XOD Activities

**Figure 3:**
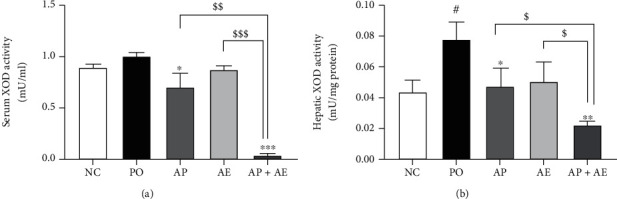
Effects of allopurinol, *Alpinia oxyphylla* seed extract (AE), and their combination on serum xanthine oxidase (XOD) activity (a) and hepatic XOD activity in hyperuricemic rats. *n* = 5 per group. NC: normal control; PO group: induced with potassium oxonate; AP: allopurinol (1 mg/kg); AE (200 mg/kg); AP + AE: AP (1 mg/kg) plus allopurinol (200 mg/kg). #*p* < 0.05 compared with the NC group; ^∗^*p* < 0.05 and ^∗∗∗^*p* < 0.001 compared with the PO group; $*p* < 0.05 and $$$*p* < 0.001 compared with the AP or AE groups.

AP and the combination of AP and AE had significant inhibitory effects on serum and hepatic XOD levels of rats (Figure 3, *p* < 0.001 compared with the PO group), decreasing them even lower than in the NC group. AE alone showed a minor effect on serum and hepatic XOD levels. Treatment with the combination significantly suppressed these levels (*p* < 0.001 compared with the PO group) and enhanced the activities of AE on serum and hepatic XOD levels (*p* < 0.001 and *p* < 0.05, compared to AE alone, respectively). These results suggested that the enhanced effects in serum uric acid levels observed after treatment with the combination of AP and AE were related to changes in serum and hepatic XOD activities.

### 3.4. Effects of the AP and AE Combination on Expression Levels of OAT1, OAT3, URAT1, and GLUT9 in Kidney Tissues

**Figure 4:**
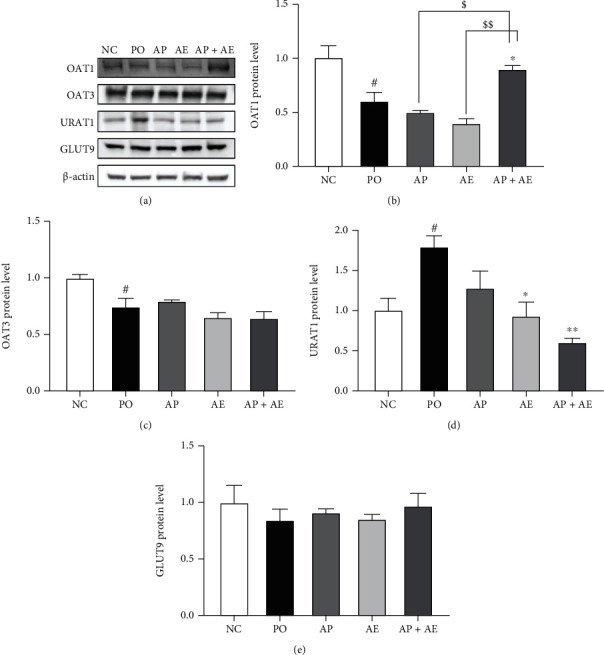
Effects of allopurinol, *Alpinia oxyphylla* seed extract (AE), and their combination on protein levels (a) of OAT1 (b), OAT3 (c), URAT1 (d), and GLUT9 (e) in the kidney tissue of hyperuricemic rats. Protein levels were normalized to beta-actin. *n* = 5 per group. NC: normal control; PO group: induced with potassium oxonate; AP: allopurinol (1 mg/kg); AE (200 mg/kg); AP + AE: AP (1 mg/kg) plus allopurinol (200 mg/kg). #*p* < 0.05 compared with the NC group; ^∗^*p* < 0.05 compared with the PO group; $*p* < 0.05 and $$*p* < 0.01 compared with the AP or AE groups.

The effects of the combination of AP and AE on OAT1, OAT3, URAT1, and GLUT9 protein levels in the kidneys of hyperuricemic rats are shown in Figure 4. We observed significantly decreased OAT1 and increased URAT1 in kidney tissues of PO rats (*p* < 0.05). Treatment with the combination of AP and AE increased OAT1, when compared to the PO group (*p* < 0.05), and increased these changes, when compared with AP or AE alone (*p* < 0.05 and *p* < 0.01, respectively). AE alone and in combination with APl decreased URAT1 protein levels (*p* < 0.05 compared to the PO group). These results suggested that the enhanced effects of the combination treatment were related to changes in renal transporter expression.

### 3.5. Effects of the AP and AE Combination on Serum and Kidney IL-1*β* Expression

**Figure 5:**
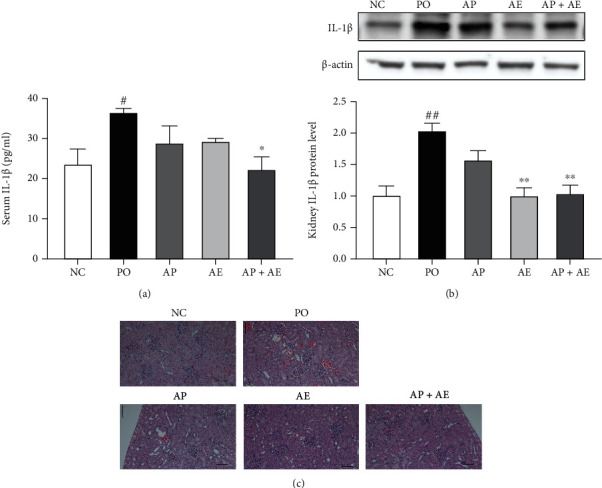
Effects of allopurinol, *Alpinia oxyphylla* seed extract (AE), and their combination on serum levels (a) and kidney protein expression (b) of IL-1*β* in hyperuricemic rats. Protein levels were measured by western blot analysis normalized to *β*-actin. (c) Renal histopathological changes (original magnification ×100) shown by hematoxylin and eosin staining. *n* = 5 per group. NC: normal control; PO group: induced with potassium oxonate; AP: allopurinol (1 mg/kg); AE (200 mg/kg); AP + AE: AP (1 mg/kg) plus allopurinol (200 mg/kg). #*p* < 0.05 and ##*p* < 0.01 compared with the NC group; ^∗^*p* < 0.05 and ^∗∗^*p* < 0.01 compared with the PO group.

We next determined whether combined treatment with AP and AE affected the expression of IL-1*β* protein in the serum and kidney (Figure 5). Increases in IL-1*β* expression were observed in the serum and kidney tissues of PO-induced hyperuricemia rats (*p* < 0.05 and *p* < 0.01, respectively). AP or AE alone had only minor effects on serum IL-1*β* levels, but treatment with the combination led to a significant decrease (*p* < 0.05). In the kidney, AE alone and combined with AP decreased IL-1*β* levels, when compared with the PO-treated group (*p* < 0.01). Mild renal tubular dilatation, dilation of Bowman's space, hemorrhage, tubular vacuolar degeneration, and inflammatory cell infiltration were observed in the kidneys of PO-induced hyperuricemic rats (Figure 5(c)). However, hyperuricemic rats treated with AE and AP showed improvements in these renal histopathological changes. These results suggested that the AP and AE combination decreased kidney inflammation in hyperuricemic rats.

## 4. Discussion

AP has been clinically used for more than 40 years and is currently the most widely used urate-lowering drug for treating chronic gout. However, AP cannot be used when allopurinol treatment fails or when the patient is hypersensitive or intolerant to the drug [19]. Although rare, AP can have life-threatening adverse effects such as hypersensitivity syndrome, in which the mortality rate approaches approximately 20% [20, 21]. We therefore combined AP with a natural plant product to enhance its therapeutic effects and reduce its potential toxicity.

*A. oxyphylla* and its constituent nootkatone are effective in treating hyperuricemia because it enhances uric acid excretion [15]. As an edible plant, the dry seed of *A. oxyphylla* is safe for human consumption as a food or medicine [22]. In our previous study, a 2-week acute toxicity test showed that AE (5,000 mg/kg given orally) did not induce any toxicity, body weight changes, or mortality during a 14-day observation period. This result indicated that the median lethal dose was more than 5,000 mg/kg. It is therefore reasonable to consider the combined use of AP and AE to lower clinically required high doses of allopurinol.

The combination of AP and AE normalized serum urate concentrations and alleviated impaired renal function by reducing serum creatinine and BUN levels. It was reported that there was a close correlation between serum urate and creatinine concentrations in gout patients [23]. Increases in serum BUN and creatinine indicate nephrotoxicity and cause renal damage in hyperuricemia [24]. Our results suggested that the addition of AE to AP therapy could prevent renal damage.

Induced inflammation by urate can stimulate kidney injury in hyperuricemic rodents [2]. Proinflammatory cytokines play important roles in various immune diseases. Although IL-1*β* production occurs in many inflammatory conditions, gout inflammation is unique because it is primarily driven by IL-1*β* [25]. Gout is caused by precipitation of uric acid from the blood into insoluble monosodium urate crystals, which form during periods of hyperuricemia and activate the NOD-like receptor family pyrin domain containing-3 inflammasome resulting in secretion of IL-1*β* [25, 26]. Thus, this study investigated IL-1*β* production in the kidney. The results showed that a combination of AP and AE reduced serum and renal IL-1*β* levels in hyperuricemia rats, suggesting that this treatment exerted anti-inflammatory and nephroprotective effects to prevent hyperuricemia from developing into gout.

To investigate the underlying mechanisms of action of the uric acid-lowering effect, we evaluated how combination treatment affected XOD activity and protein expression of renal transport in the hyperuricemic rat. The AP and AE combination reduced serum and hepatic XOD levels even lower than measured in the NC group, which likely reduced the sources of urate generation. Western blot results showed that the combination of AP and AE downregulated the expression of renal URAT1 protein while upregulating that of renal OAT1. The net effect was increased renal excretion of urate. Recently, it was reported that supplementation of extracted stevia residue with AP attenuated hyperuricemia via inhibiting the production of urate (XOD inhibition) and inflammation without any side effects in animal model studies [27]. The results of the present study showed that a combination treatment with AP and AE provided a dual mechanism for lowering serum urate concentrations by increasing excretion via regulation of URAT1 and OAT1 and reducing urate production via XOD inhibition.

AP is better known for the management of hyperuricemia. The rationale for use of AP in hereditary renal hypouricemia patients was to decrease the generation of UA, thus, decreasing the filtered UA load and lowering the risk of precipitation of UA in tubules. With this approach, it was reported that use of AP effectively prevented recurrence of renal injury in this patient [28]. Thus, use of the AE and AP combination in patients with common dysfunctional URAT1 variants should prevent repeat symptoms or recurrent acute kidney injury.

It was also reported that common dysfunctional variants of the high capacity ATP-binding cassette urate transporter (ABCG) gene were major risk factors of gout and hyperuricemia, involving a decrease in urate excretion [29]. In a previous study, ABCG2 protein levels were changed by AE (data not shown). The ABCG2 risk alleles have been associated with AP resistance, and it is possible these variants lead to relative hyperuricemia even with urate-lowering AP therapy in patient with gout [30–32]. Thus, use of AE combined with AP should prevent hyperuricemia in patients who are inadequate AP responders.

The active metabolite of AP, oxypurinol, is a substrate of URAT1. AE may inhibit the uptake (tubular reabsorption) of oxypurinol through URAT1 after its excretion in urine, thus reducing the blood exposure of oxypurinol. However, further study is needed to evaluate the potential pharmacodynamic drug-drug-interactions between the selective urate excretion inhibitor, AE, and the XO inhibitor, AP (and its active moiety, oxypurinol).

Our results suggest that there may be medicinal value in combining low dose AP with AE for the treatment of hyperuricemia, especially in patients with renal insufficiency. The commonly known dose-related side effects of AP could be attenuated with the addition of AE. However, safety and long-term studies of the combination for clinical use should be further investigated.

## 5. Conclusions

We determined whether *A. oxyphylla* potentiated the effect of AP on serum uric acid concentrations in a hyperuricemic rat model induced by PO. Combination treatment of AP and AE provided a dual mechanism for lowering serum uric acid concentrations, involving increased excretion via regulation of URAT1 and OAT1 and reduced urate production via XOD inhibition. These results suggest that the combination of AP and AE may have clinical value for treating hyperuricemia.

## Data Availability

The data used to support the findings of this study are included within the article.

## Supplementary Materials

Supplementary Materials Figure S1: Preliminary dose response study of allopurinol and *Alpinia oxyphylla* seed extract.
