# Supplementary material for: Synergistic Impacts of Alpinia oxyphylla Seed Extract and Allopurinol against Experimental Hyperuricemia
Source: Biomed Res Int. 2022 Jun 11;2022:2824535. doi: 10.1155/2022/2824535 (PMC9206559; doi:10.1155/2022/2824535)
Supplement: Supplementary Materials — Figure S1: Preliminary dose response study of allopurinol and Alpinia oxyphylla seed extract. [file 2824535.f1.docx]

**SUPPLEMENTARY MATERIALS**

**Materials and Methods**

**Hyperuricemia induction and sample treatment**

To determine the dose for AP and AE treatment, the rats were randomized divided into the following 9 groups (n=5/group): (1) normal control group (NC), (2) PO-treated hyperuricemia group (PO), (3) PO + 1 mg/kg AP group, (4) PO + 2 mg/kg nootkatone group, (5) PO + 100 mg/kg AE, (6) PO + 200 mg/kg AE, (7) PO + combination of 1 mg/kg AP and 100 mg/kg AE group, (8) PO + combination of 2 mg/kg AP and 100 mg/kg AE, and (9) PO + combination of 1 mg/kg AP and 200 mg/kg AE group. The 150 mg/kg PO in 0.5% CMC-0.1 M sodium acetate was injected intraperitoneally to rats within groups (2)-(9) to induce hyperuricemia. Groups (1) and (2) received the vehicle. In the experiments, AP and AE were administrated orally, 1h after PO injection. Blood from the rats were collected 2 h following AP, AE, or AP+AE administration, and the serum was separated by centrifugation (3,000×g, 15 min, 4°C). Urate levels from serum were determined using uric acid assay kit (Abcam, Cambridge, UK).

**Fig. S1. Preliminary dose response study of AP and AE**
